# Plight of Dentistry in Pakistan

**DOI:** 10.12669/pjms.36.3.2278

**Published:** 2020

**Authors:** Shaukat Ali Jawaid

**Affiliations:** Pakistan Journal of Medical Sciences, Karachi - Pakistan

Oral health and dental care are scarcely mentioned in the reports on health care system in Pakistan and many countries abroad e.g. Canada. According to reports it was Goldsmith and Ivory Tuner Paul Revere who constructed the false teeth in Boston. In those days tooth decay and toothaches were considered to be an inevitable parts of life and looking after the teeth was considered as mechanical concern. Chapin Harris is reported to have lead the efforts to elevate this trade to a profession. Dental students learned the mechanism of drilling and filing teeth and constructing dentures and they also learned to perform dental extractions. At that time oral cavity was considered as the seat of most of the illnesses, hence removal of the teeth was most often recommended. Later it was realized that dentistry can no longer be accepted as mere tooth technology and it has to be developed as a specialty in line with developments of other medical disciplines as specialization was transforming many aspects of Western medicine. 1 As an act of defiance, the dentist and dental X-ray innovator C. Edmund Kells refused to operate upon physicians’ instructions and stood up for tooth preservation in 1920.^1^ Since then dentistry is operating in a different domain from the medical profession. Medical and Dental schools also remained separate. This autonomy of dentistry was applauded by the Journal of the American Dental Association. However, there have been calls for closer ties between dental and medical professions as dentists were not interested in looking at other parts of the body while the physicians never look at the mouth.

Unfortunately for dentistry in Pakistan, it has never gained its proper place and recognition as it remained under the shadow of Medical profession. In addition, it lacks professional and dedicated leadership which can take up the cause of this important discipline and convince the health planners in Pakistan of its usefulness and importance. Dentistry is also a very paying specialty. Cost of treatment is very high and it is also a very delicate and difficult surgery as one has to operate in the oral cavity which demands extreme care to ensure that the patient is not harmed. Setting up a dental practice also involves lot of initial investment which is not the case with many other specialties of medicine.^2^

Dentistry in America gained importance and succeeded in getting due recognition because of the high quality of dental journalism. The first dental journal, American Journal of Dental Science was published in 1839 while index to dental literature was published in 1921. Evolution of dental journals was closely linked to trade house publications which later emerged as independent scientific literature. This helped gain dentistry a professional status. Dentistry in United States did not become a profession till 1840 and its journey from mechanical trade to a professional status is very fascinating. First Dental School in USA Baltimore College of Dental Surgery was established in 1840. Thus dentistry got separated from Medicine and was recognized as a specialty in its own right.^3^ Therefore it appears that dental journalism played a critical role in promoting dentistry as a profession in the United States. Dental Journalism made its beginning in USA and in 1883, twenty dental journals were being published in United States. By 1919 its number increased to forty five. From 1839 to 1939, many dental journals were started and then ceased publication. In 1919 Dr. William Bebb in his bibliography had included articles from 50 dental journals and by 1923, American Dental Association had 33,500 members.^3^

Later, many dental journals ceased publication because of lack of finances. They failed to find the necessary resources. While the Trade Journals did fill the need for dental literature until the dental profession was large enough, got interested and could afford independent journals which could grow and survive. Most of the dental journals in USA ceased to exist or were sold to dental manufacturers who published them profitably. American Journal of Dental Science started in 1839 was published at a loss for twenty years and later sold to dental dealers in Boston. It was revived time and again till finally ceased publication in 1909. New York Dental Recorder and many other dental journals were also sold to dental manufacturers. Some of these dental journals could not get any sponsor, hence ceased publication. Dental Cosmos a dental newsletter started in 1847 was merged with another dental journal in 1936 and is now known as Journal of American Dental Association.^3^ All these developments were the result of disorganization and lack of leadership within dentistry from 1839-1939. It was pointed out that dental profession in USA is a mob which lacks inspiration, discipline and unity conferred through an effective organization. Unfortunately, all the above diseases are very much prevalent in dental profession in Pakistan today and it has no effective spokesman. Emergence of more good quality dental journals ensure independence of dentistry from Medicine. Exactly the same situation can be seen in Pakistan today in 2020, something where USA was in 1840. It is the dental journals which will give more space and facilitate reporting of new materials, procedures and treatments which won’t get proper place in medical journals. Even in 1840 it was felt in USA that none of the dental journals matched and fulfilled the needs of dental profession. Prior to 1840, dentistry in USA was also a mechanical trade. As dental journalism grew, dentistry became better organized, more egalitarian profession. Emergence of more dental journals encouraged cooperation, contributed to further evolution of dentistry from a scientific and organizational standpoint.

Coming to Pakistan, in the past there used to be just one dental institution i.e. de’Montmorency College of Dentistry at Lahore. Later dental sections were established with Khyber Medical College at Peshawar. Liaquat Medical College Hyderabad and Nishtar Medical College Multan. The first private dental college was established at Baqai Medical University at Karachi in 1992. During the last two decades, we have seen a mushroom growth of medical and dental colleges in the public as well as private sector. Most of the medical universities as well as private medical colleges have now also established dental colleges. However, most of those who have established these institutions are lacking the critically required qualified faculty for providing quality student teaching and training and are more interested in making money. These institutions are not planned based on factors like population skill requirements, job market and oral health goals. Unfortunately, these are rather established, as they are a good business proposition. Currently there are fifty five dental schools in Pakistan both in public and private sector of which twenty six are in Punjab, eighteen in Sindh, ten in KPK and one in Baluchistan. A few more dental colleges are likely to be established soon as they are waiting government permission after having developed the requisite set-up. However, there is acute shortage of real academicians in the dental profession. We do have some very distinguished dentists, well trained, experienced and highly qualified but they are not in a position where they can make some difference as a vast majority of dental institutions are headed by business owners, who have no or least interest in academics and research.^2^ Quite a few brilliant dental researchers and academicians were lost to the emerging new discipline of medical education due to lack of enough opportunities for post-graduation and government jobs.

Specialization in Dentistry had been there in Paksitan since long with the Punjab University accredited specialist dental program of MDS (Major Dental Qualification) in many dental specialties. However, most senior dental teachers remained reluctant to develop their own profession/ specialties and had remained interested in only giving the specialist qualification of MDS degrees to the very few who were their favourite with the result that it never became popular and had to be discontinued. It was in 90s that the College of Physicians & Surgeons Pakistan started a specialist training programme in dentistry which was first named as Membership of Faculty of Dentistry (MFD) in major clinical disciplines of dentistry. Late Prof. Sultan Farooqui former President of CPSP deserve special mention for having taken up the cause of dentistry and helped the start of Fellowship in various dental discipline’s. It was in 1994 that the Dental Faculty at CPSP planned FCPS programmes in six dental disciplines. The identified prospective FCPS training supervisors were invited to a meeting by the CPSP held at Karachi on July 15^th^ 1994 who finalized the format of FCPS examination, training requirement, SYLLABI and units which could provide this training were also identified. Before the Year 2000, a combined dental faculty existed at the CPSP however, later separate specialist faculties in various dental disciplines were established.^4^

Leadership of the dental profession in Pakistan is practically with the mechanical trade i.e. manufacturers of dental instruments, dealers in dental equipment and dental material. At every dental conference billions of rupees are spent and the number of stalls put up in the exhibition is over one hundred representing all segments of dental equipment, instruments, materials and not to forget the other players in oral health i.e. manufacturers of tooth brushes and toothpaste etc. The venue gives the impression of a “Dental Bazar” and less of a dental conference.^2^ This was exactly the case in United States in 1840 hence it won’t be an exaggeration to say that dentistry in Pakistan at present is where dentistry in United States used to be prior to 1840.^3^ Even historically dentistry has gone through different phases all over the world before it got recognition as an independent separate discipline of medicine. Setting up, running and managing a dental school had never been easy in the past too. Writing in an Editorial, Journal of American Dental Association in 1967 referred to the decision taken by St. Louis University to discontinue its dental school. It further stated that in 1960, twenty nine of the forty seven dental schools almost two thirds were under the auspices of private universities. In 1967, out of the fifty functioning dental schools, almost 50% were in private universities while all the new and developing dental schools were state-supported. Private universities displayed a lack of interest in entering the field of dental education due to various reasons.^5^ The editorial made a point that dental education deserves financial support of the community. The state must recognize its obligation to provide dentists who can take care of the oral health of the population.

It was reiterated that universities and other academic institutions should reduce the administrative workload and provide appropriate level of mentoring which will pay rich dividends both in teaching as well as research. Recruiting the new generation of academicians is not difficult provided they are offered mentorship and support.^5^ Carol Tran writing about the plight of Australian Dental Schools reported that” it was extremely disappointing to see the programmes and staff members come and go. Sometimes one does become disheartened and disillusioned in academic. However, there are great rewards when one achieves a good teaching outcome or is awarded with a grant”. He further stated that life can be much more balanced in clinical practice than in academia which might include planning a curriculum and teaching hundreds of students. Academics is not for the faint hearted since the risk of burnout is very high, especially for the young academic staff.^6^.^7^ The authors urged the dental schools to support their junior staff members both in teaching and research thus supporting the suggestion of Bolton.^8^

A critical look at the dental literature will reveal that despite numerous hardships and hurdles, dentistry has made tremendous progress over the last few decades and now it has numerous sub-specialties functioning not only overseas but also in Pakistan. American Dental Association recognizes about a dozen dental sub-specialties which includes Dental Public Health, Endodontics, Oral and Maxillofacial Pathology, Oral and Maxillofacial Radiology, Oral and Maxillofacial Surgery, Orthodontics, Prosthodontics, Restorative and Cosmetic Dentistry and Implant dentistry. In Pakistan, CPSP now offers Fellowship in five dental sub-specialties which include Operative Dentistry, Oral and Maxillofacial Surgery, Orthodontics, Periodontology and Prosthodontics. However, despite all this, dentistry has failed to get professional status and lacks independent Dental Council in Pakistan, which it deserves. So far just one or two dental surgeons have been part of the deceased PM&DC, failing to voice the critical concerns of dentists and dentistry as a professional specialty and its pivotal role in the overall health of the Pakistani population. Now PM&DC has been replaced with Pakistan Medical Commission (PMC) effectively eliminating “D” which used to represent dentistry. Therefore, most of the decisions pertaining to dentistry which were taken by medical professionals have failed to promote and project dentistry in its correct manner in Pakistani society. It may be related to the absence of dental academicians or linked with the quality and standard of dental journalism, which is dependent on the amount, and quality of dental research being carried out in Pakistan- an area which is almost barren and highly disappointing. We have just two dental journals of varying quality and none of them has been able to achieve an Impact Factor which is one of the important criteria to judge the quality and standard of a journal. Few dental specialists’ societies including Pakistan Prosthodontics Association (PPA) and Pakistan Association of Orthodontics (PAO) have launched their journals but have the same reputation.

In Pakistan dental profession is lacking curiosity and interest in academics or research is the least priority. Except a few, not many bother to write or do research, hence dental journalism has not yet developed. However, overseas, by 1900 the emphasis had shifted towards prevention and treatment of dental diseases while before 1840, dentistry was considered a specialty of medicine and physicians used to be Generalists. Establishment of American Society of Dental Surgeons caused permanent rift between Dentistry and Medicine and the dentists desire to control their own literature, widened this difference between Dentistry and Medicine. However, no such danger exists in Pakistan for the time being. In USA it was through dental journals that Dentistry changed from a Mechanical Trade to a Profession which also lead to the formation of American Association of Dental Editors (AADE).

In view of the above, it is high time that Pakistan Dental Association or leadership in dentistry wakes up. The way they have been running its affairs has failed to help the discipline of Dentistry to get due recognition and respect. They must give up personal internal politics, instead select dental academicians with a vision and foresight who can convince the authorities on the usefulness of having a separate Pakistan Dental Council to promote and safeguard the interest of dentistry as a discipline. In the 80s Prof. Dr.M.A. Soofi (late) did try for a separate Pakistan Dental Council but he failed to get support from his colleagues. Dr.Arif Alvi presented a draft of Dental Act to the then Prime Minister of Pakistan Mr. Mohammad Khan Junejo without any positive outcome. The PDA then also tried to get the Dental Act enacted through Senator Javed Jabbar during General Pervez Musharraf’s Government but again failed. Now it is a very happy coincidence that Dr.Arif Alvi himself is occupying the highest coveted constitutional post of President of Pakistan and his own party is in power. It would be fitting that the profession, which gave him recognition, be promoted to an independent body in his time of governance. It should not be difficult for him to persuade his own Government for uplifting the professional status of dentistry and with that the plight of hundreds of thousands of professionals associated with dentistry including, dental hygienist, dental technicians, dental therapist, dental academicians and researchers. In addition, this will further the oral health quality and education of the Pakistani nation and reduce the burden of oral health care treatments for the government.

In the meantime, the dental profession must also set its house in order. Let those with academic and research interests as well as leadership qualities be appointed to the coveted posts of Principal and Deans of Dental Schools. They should revise the curriculum, improve the teaching and training programmes bringing them at par with international standards. Dental institutions in Pakistan should also benefit from the expertize of dental academicians of Pakistani origin who are occupying coveted posts in dental institutions overseas by inviting them as visiting faculty. Give them due respect and many of them will be glad to help their motherland. A sincere effort can bring about a positive change improving the plight of dentistry and dental profession in Pakistan.
